# Consumption Trends of Antibiotics in Brazil During the COVID-19 Pandemic

**DOI:** 10.3389/fphar.2022.844818

**Published:** 2022-03-21

**Authors:** Fernando de Sá Del Fiol, Cristiane de Cássia Bergamaschi, Isaltino Pereira De Andrade, Luciane Cruz Lopes, Marcus Tolentino Silva, Silvio Barberato-Filho

**Affiliations:** School of Pharmacy, University of Sorocaba, Sorocaba, Brazil

**Keywords:** Azithromycin, COVID-19, Pharmacoepidemiolgy, Antimicrobial resistance (AMR), Hydroxychloroquine (HCQ)

## Abstract

**Background:** In 2019, a new type of coronavirus emerged and spread to the rest of the world. Numerous drugs were identified as possible treatments. Among the candidates for possible treatment was azithromycin alone or in combination with other drugs. As a result, many clinicians in Brazil have prescribed azithromycin in an attempt to combat or minimize the effects of COVID19.

**Aim:** This study analyzed the sales data of the main antibiotics prescribed in Brazil to verify the change in consumption trends of these drugs during the COVID-19 pandemic.

**Methods:** This is an interrupted time series that analyzed antimicrobial sales data between January 2014 and July 2021, publicly accessible information obtained from the Brazilian government’s website. Monthly means of “defined daily doses of DDDs” (DDDs per 1,000 inhabitants per day) of antibiotics were compared by analysis of variance, followed by the Dunnett Multiple Comparisons Test. Monthly trend changes in antibiotic use were verified using Joinpoint regression.

**Results:** Amoxicillin (31.97%), azithromycin (18.33%), and cefalexin (16.61%) were the most sold antibiotics in Brazil during the evaluation period. Azithromycin consumption rose from 1.40 DDDs in February 2020 to 3.53 DDDs in July 2020. Azithromycin sales showed a significant increase in the pandemic period [Monthly Percent Change (MPC) 5.83%, 95% 1.80; 10.00], whereas there was a fall in amoxicillin sales (MPC −9.00%, 95% CI −14.70; −2.90) and cefalexin [MPC-2.70%, 95% (CI −6.30; −1.10)] in this same period.

**Conclusion:** The COVID-19 pandemic changed the pattern of antibiotic consumption in Brazil, with a decrease in the use of amoxicillin and cefalexin and an increase in the consumption of azithromycin.

## Introduction

In 2019, a new type of coronavirus emerged in China and spread to the rest of the world, causing the World Health Organization to decree that there was a new pandemic in March 2020 (Cucinotta and Vanelli, 2020). The transmission of the new virus occurs through the respiratory route, with symptoms including fever, cough, runny nostrils, and atypical pneumonia; the latter is largely responsible for worsening the clinical condition of affected patients. Changes in taste and smell also completed the clinical condition of most patients affected by the new infection (Group, 2021).

With a mortality rate that ranged from up to 5% at the beginning of the pandemic to approximately 2.2% at the beginning of 2021 (Mallah et al., 2021), health authorities sought and continued seeking alternatives to prevent and combat the new coronavirus. Measures of social distancing, use of masks, closing schools and stores, and using hand sanitizer were the main measures adopted at the beginning of the pandemic, intending to reduce transmission rates, while there was no effective treatment or a vaccine able to reduce transmission and mortality rates among those affected (Morawska and Cao, 2020).

In 2020, with the exponential increase in the number of cases and the number of countries affected and, until that moment, without a vaccine available for everyone that could reduce the effects of the new virus, several treatment proposals were made based on re-purposed drugs that already had their safety established. Numerous drugs were identified as possible treatments, being the reason for many systematic reviews and meta-analyses (Kim et al., 2020; Budhathoki et al., 2021; Diaz-Arocutipa et al., 2021; Kumar et al., 2021).

Amongthe candidates for possible treatment, an association between azithromycin and hydroxychloroquine has been widely propagated and studied in combating new infections (Ghazy et al., 2020; Kim et al., 2020; Prodromos and Rumschlag, 2020; Siemieniuk et al., 2020; Kashour et al., 2021; Million et al., 2021). The anti-inflammatory and immunomodulatory properties of this macrolide directed its use as a possible candidate for the treatment of COVID-19 (Zarogoulidis et al., 2012). Studies, so far, do not show efficacy in the use of azithromycin associated with other drugs, in the treatment of COVID-19 (Cavalcanti et al., 2020; Furtado et al., 2020; Ghazy et al., 2020; Rosenberg et al., 2020; Fiolet et al., 2021)Using antibiotics, in a prophylactic or even therapeutic format, in addition to direct adverse effects, can lead to the emergence of resistant bacterial specimens in the medium and long term, as recently demonstrated (Doan et al., 2020).

The literature is scarce in studies that have analyzed the consumption of the main antibiotics prescribed in Brazil, with emphasis on azithromycin, prescribed throughout the national territory, during the pandemic. This study analyzed the sales data of the main antibiotics prescribed in Brazil to verify the change in consumption trends of these drugs during the COVID-19 pandemic.

## Methods

### Study Design

An interrupted time series was used to analyze the consumption trends of amoxicillin, azithromycin, and cefalexin antibiotics (outcome of interest) during the COVID-19 pandemic (exposure of interest).

### Setting and Study Size

Pharmacies and drugstores in Brazil have been required to register the number of antibiotics sold monthly in the National System of Controlled Products Management (known by the acronym SNGPC) since 2013 (ANVISA and Sanitária, 2011). Monthly sales volume data were collected between January 2014 and July 2021. However, these data only became publicly available in November 2020 (Brazil, 2021). Monthly sales volume data were collected between January 2014 and July 2021.

### Data Sources, Measurement, and Variables

Data were collected in.csv format, taken to a data server, and then the following variables were extracted: name of the active ingredient, trade name, and respective presentations, patient’s age, prescriber, and location of sale (city and state).

Based on the number of commercial presentations of each antibiotic sold and the concentration of an active ingredient in each commercial presentation, the number of defined daily doses (DDDs)/1,000 inhabitants/day for each antibiotic was calculated, as recommended by the World Health Organization (WHO, 2021). Consumption was expressed in DDDs/1,000 inhabitants/day.

### Statistical Methods

To compare the monthly mean DDDs per 1,000 inhabitants per day of antibiotics, analysis of variance (ANOVA) was used, followed by the Dunnett Multiple Comparisons Test (Graph Pad Instat (Version 3.05). Antibiotic consumption was compared between the studied years.

To assess the changes in monthly trends in the use of the antibiotics studied, we applied the Joinpoint regression, a statistical method used to identify the best-fitting points if there is a statistically significant change in a trend, assessing changes in time series data (Brodeur et al., 2021). The Joinpoint Regression Program was used (Version 4.9.0.0. March 2021; Statistical Research and Applications Branch, National Cancer Institute).

## Results

Between January 2014 and July 2021, approximately 800 million packages containing antibiotics were sold in pharmacies and drugstores in Brazil. Amoxicillin, azithromycin, and cefalexin accounted for 67% of the sales (Table 1).

**TABLE 1:** Top selling antibiotics in Brazil between January 2014 and July 2021 and their market share (%).

Drugs (ATC)	Packages sold	%	Cumulative %
Amoxicillin (J01CA04)	179,518,563	32.0	32.0
Azithromycin (J01FA10)	102,948,874	18.3	50.3
Cefalexin (J01DB01)	93,291,489	16.6	66.9
Ciprofloxacin (J01MA02)	67,369,118	12.0	78.9
Levofloxacin (J01MA12)	39,009,875	7.0	85.9
Sulfamethoxazole and trimethoprim (J01EE01)	20,763,544	3.7	89.9
Metronidazole (J01XD01)	19,924,333	3.6	93.1
Ceftriaxone (J01DD04)	15,331,526	2.7	95.8
Cefadroxil (J01DB05)	12,034,288	2.1	98.0
Norfloxacin (J01MA06)	11,367,427	2.0	100.0
Total packages sold	561,559,037	100.00	

**Notes**: ATC, anatomical therapeutic chemical classification.

### Antibiotic Consumption Trends Before and During COVID-19

**FIGURE 1:**
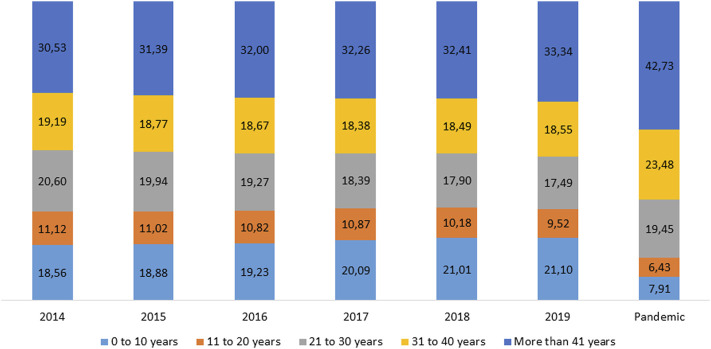
Defined Daily Dose per 1,000 inhabitants per day trend of amoxicillin, azithromycin, and cefalexin before and after COVID-19 in Brazil per month (monthly moving average for 5 months).

Figure 1 shows the DDD per 1,000 inhabitants per day for the three antibiotics most consumed before and during COVID-19 per month. In March 2020 (before COVID-19 started in Brazil), amoxicillin and azithromycin had annual seasonality, and cefalexin had regular consumption. Between 2014 and 2016, azithromycin was consumed more than amoxicillin. This consumption equaled in 2017–2018, and amoxicillin passed the consumption in 2019, but this scenario was discontinued after COVID-19. Azithromycin consumption increased from 1.40 DDDs in February 2020 to 3.53 in July 2020. Amoxicillin slowed its trend to 0.99 DDDs in April 2020, and cefalexin also reduced its consumption somewhat.

The trend of antibiotic consumption is also observed when analyzing the data by year (Table 2). Azithromycin showed a downward trend until 2019, and its consumption increased during COVID-19. Amoxicillin was in an upward trend and showed a substantial decline after COVID-19. During the pandemic period, amoxicillin, azithromycin, and cefalexin showed statistically significant differences compared to some previous years, as showed in Table 2 (*p* < 0.01).

**TABLE 2:** Defined Daily Dose per 1,000 inhabitants per day (Mean), Standard Deviation (SD) and *p* value of amoxicillin, azithromycin, and cefalexin before and during Pandemic period in Brazil, per year.

Year	Amoxicillin			Azithromycin			Cefalexin		
	Mean	SD	p	Mean	SD	p	Mean	SD	p
2014	1.57	0.26	0.98	2.24	0.34	0.72	0.44	0.05	<0.01*
2015	1.72	0.17	0.31	2.28	0.45	0.85	0.47	0.06	<0.01*
2016	1.84	0.31	0.02	2.27	0.47	0.81	0.52	0.04	<0.01*
2017	2.02	0.26	<0.01*	2.04	0.24	0.09	0.46	0.03	<0.01*
2018	2.02	0.25	<0.01*	1.99	0.29	0.04*	0.43	0.02	<0.01*
2019	2.15	0.27	<0.01*	1.82	0.31	<0.01*	0.43	0.02	<0.01*
Pandemic period	1.49	0.35	—	2.49	0.64	—	0.36	0.04	—

**Notes**: *Indicate significant differences (at *p* < 0.05) when compared to Pandemic period (January 2020 to July 2021).

**FIGURE 2:**
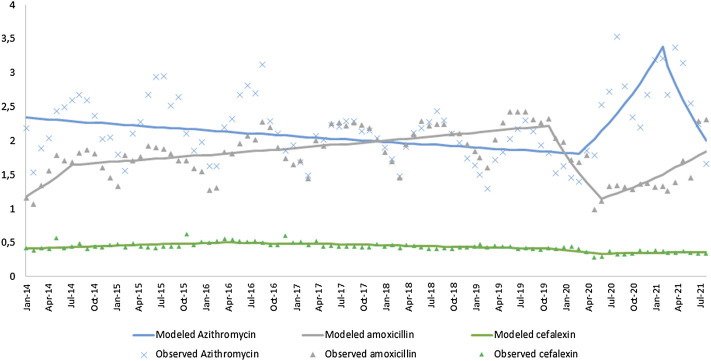
Observed and modeled monthly consumption (DDDs) of the antibiotics studied, with changes in trends (Joinpoint).

The joinpoint analysis available in Table 3; Figure 2 revealed three significant joints for amoxicillin sales (July 2014 October 2019, and May 2020), two for azithromycin sales (February 2020 and January 2021), and three for cefalexin sales (March 2016 October 2019, and May 2020). Amoxicillin sales remained stable through October 2019 but declined significantly after the onset of the pandemic (MPC −9.00, 95% CI [−14.70; −2.90]). The decrease in amoxicillin sales soon after the beginning of the pandemic can be explained by social isolation and the decrease in consultations with doctors’ offices, as some studies have already shown. (King et al., 2021; Ha et al., 2022; Norman et al., 2022). Use of face masks and social distancing also contributed to the decrease in the transmissibility of respiratory diseases and the consequent use of antibiotics.

**TABLE 3:** Joinpoint analysis for amoxicillin, azithromycin, and cefalexin sales in Brazil by month, January 2014 to July 2021.

	Length time	Month range	MPC tendency	MPC % (95% CI)
Amoxicillin	Jan-14 to Jul-14	6	↑	5.69 (−1.00; 12.80)
	Jul-14 to Oct-19	65	↑	0.48 (0.30; 0.70)
	Oct-19 to May-20	7	↓	−9.00 (−14.70; −2.90)
	May-20 to Jul-21	14	↑	3.41 (1.60; 5.30)
Azithromycin	Jan-14 to Feb-20	75	↓	−0.35 (−0.50; −0.20)
	Feb-20 to Jan-21	11	↑	5.83 (1.80; 10.00)
	Jan-21 to Jul-21	6	↓	−8.33 (−15.8; −0.20)
Cefalexin	Jan-14 to Mar-16	27	↑	0.75 (0.30; 1.20)
	Mar-16 to Oct-19	44	↓	−0.46 (−0.70; −0.30)
	Oct-19 to May-20	7	↓	−2.70 (-6.30; −1.10)
	May-20 to Jul-21	14	↑	0.40 (−0.70–1.50)

Joinpoint regression: * MPC, Monthly percent change is significantly different from zero at alpha = 5%.

Among azithromycin sales, joinpoint analysis also revealed a significant increase in the pandemic period (MPC 5.83, 95% CI 1.80; 10.00), in contrast to the earlier and more recent decline period.

The increase in azithromycin sales found in the present study occurs precisely when the first cases of COVID-19 appear in Brazil and the first treatment proposals involving the antibiotic begin to be published. (Gautret et al., 2020; Gilzad-Kohan and Jamali, 2020; Million et al., 2020; Wu et al., 2020). There was a great demand for Azithromycin in Brazilian pharmacies, with a sustained increase of 5.83% per month, starting in February 2020 and continuing until January 2021. It is very likely that a large part of this demand has occurred by people who would like to have the antibiotic in their homes so that they could use it, if necessary. In February 2021, with the start of vaccination in Brazil and with more clarity about the ineffectiveness of its associated or isolated use (Cavalcanti et al., 2020; Furtado et al., 2020; Ghazy et al., 2020; Rosenberg et al., 2020; Fiolet et al., 2021), sales started to fall by about 8% per month.

Among cefalexin sales, the joinpoint showed a stable trend, except during the pandemic period (MPC −2.70, 95% CI [−6.30; −1.10]).

**FIGURE 3:**
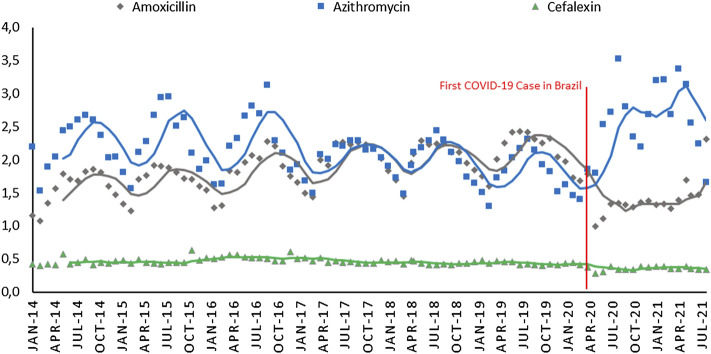
Distribution in percentages of age groups of azithromycin consumption prescribed, in Brazil, between January 2014 and July 2021.

When considering the patient’s age, azithromycin sales changed significantly over time. Figure 3 shows the azithromycin sales stratified by patient age group. Before the pandemic, prescriptions were aimed at children (0–10 years) and older adults (>40 years). After the pandemic, this trend changed, with more prescriptions for middle-aged adults (31–40 years old) and older adults and fewer prescriptions for children.

## Discussion

Amoxicillin, azithromycin, and cefalexin were the most commonly used antibiotics in Brazil during the period evaluated. There was an important seasonal variation for both amoxicillin and azithromycin, with an increase in DDD in the colder months of the southern hemisphere (June, July, and August). Both amoxicillin and azithromycin have been widely used for respiratory infections, which occur more frequently in the coldest months of the year (Young et al., 2020). The three most prescribed antibiotics in Brazil (cephalexin, azithromycin and amoxicillin) accounted for 2/3 of all prescriptions since 2014. Azithromycin and amoxicillin used as a first choice for respiratory infections together accounted for half of the antibiotics prescribed in the country (Laopaiboon et al., 2015). For cephalexin, with main indications for pregnant women and/or urinary infections, it represented only 16% of prescriptions.

The results showed an increase in the consumption of azithromycin during the pandemic, with statistically significant decreases in the consumption of amoxicillin and cefalexin during the pandemic. On the one hand, the decrease in the use of amoxicillin and cefalexin can reduce the levels of resistance to pathogens (Bruyndonckx and Coenen, 2021). An increase in azithromycin use will certainly have consequences on the resistance levels of pathogens causing acute respiratory infections (Bergman et al., 2006; Schroeder and Stephens, 2016). The significant increase (5.8% per month) in the consumption of azithromycin, especially at the beginning of the pandemic, showed a rush of the population towards a supposed “treatment.” Without the wide availability of a vaccine, the population sought drugs that could treat their COVID-19 infection, and reduce its intensity or lethality, without scientific proof.

During the pandemic, in Brazil, there was a decrease in prescriptions for amoxicillin and cefalexin. Cephalexin sales data remained stable throughout the pre-pandemic period (2014–2019), with small variations in DDDs (0.43–0.52), however, during the pandemic, sales data showed a significant reduction for about 0.36 DDDs. These decreases are clearly explained by the decrease in bacterial respiratory infections during the pandemic. Other authors found the same phenomenon, attributing the drop in respiratory infections (not COVID-19 infections) during the pandemic to social distancing (for example, schools closing), the use of face masks, decreased visits to doctors’ offices and emergency services, and measures of more restrictive health protocols during the pandemic (Kuitunen et al., 2020a; Kuitunen et al., 2020b; Chiapinotto et al., 2021; Huang, 2021). All these measures contributed to the decrease in the transmissibility of respiratory pathogens, with a consequent decrease in the number of infections and use of these antibiotics. The COVID-19 pandemic, despite all the problems it brought to humanity, also showed that appropriate health behaviors are important tools in combating infections, and consequently, the use of antibiotics and their consequences.

The increase in the overstated consumption of azithromycin has also occurred in other countries that have evaluated its use in the hospital setting (Castro-Lopes and Correia, 2021; Grau et al., 2021; Grau and Hernández, 2021; Sulis et al., 2021), showing similar increases to the present study, with a 2-fold higher consumption of azithromycin in the pandemic period than in previous periods.

Data referring to age group support the hypothesis that the consumption of azithromycin was directly related to the pandemic, as children, being less susceptible to COVID-19 infection (Gallo Marin et al., 2021; O'Driscoll et al., 2021), were those that showed a proportional decrease in consumption when compared to the age groups of adults (20 years or more). The same result was found by (Sulis et al., 2021) which showed that the increase in azithromycin sales in India during the pandemic also did not reach the lowest age groups.

The sudden increase in azithromycin sales to adults in Brazil can be explained by a search for a possible treatment for COVID-19. After the start of vaccination, these sales fall at the same rate as they rose. The consequences of this inappropriate use could impact the increase in resistance indicators of respiratory pathogens, the main target of this antibiotic.

Although Brazil has significantly advanced with the SNGPC system, as of 2013, controlling the sale of antibiotics only with the presentation and retention of the prescription, there is still much to be done in terms of pharmacologically educating antibiotic prescribers to use this class of drugs in a rational way to avoid the precocity of its ineffectiveness.

The measures forcibly adopted by the population (use of masks, social distance, hand sanitizer) also showed us that it is possible to reduce respiratory infections as hygiene measures and not just pharmacological measures. A smaller number of respiratory infections reflect a lower use of antibiotics, which equals the decrease in antimicrobial resistance indices.

### Study Limitation/Strength

Our study was able to evaluate about 560 million antibiotics sold in pharmacies throughout Brazil for about 8 years, which guarantees a significant sample of how antibiotics are prescribed in the country and how the pandemic has changed this scenario. Data were collected from all states in the country. It is also important to highlight that, unfortunately, the Brazilian system that provides the data is not able to show the indication of each prescription, that is, which infection is being treated with that antibiotic, which could further deepen the analysis and the trends in the use of antibiotics in the country, ensuring more adequate public policies for the judicious use of antibiotics.

## Conclusion

The COVID-19 pandemic changed the pattern of antibiotic consumption in Brazil, with a decrease in the use of amoxicillin and cefalexin and an increase in the consumption of azithromycin. The unnecessary use of azithromycin may result in higher levels of resistance to respiratory pathogens, in addition to a false sense of protection against Covid. Actions worldwide to combat the inappropriate use of antibiotics are increasingly necessary so that the next pandemic is not caused by a multi-resistant bacterium.

## Data Availability

Publicly available datasets were analyzed in this study. This data can be found here: https://dados.gov.br/dataset/venda-de-medicamentos-controlados-e-antimicrobianos-medicamentos-industrializados.
